# Press-pulse: a novel therapeutic strategy for the metabolic management of cancer

**DOI:** 10.1186/s12986-017-0178-2

**Published:** 2017-02-23

**Authors:** Thomas N. Seyfried, George Yu, Joseph C. Maroon, Dominic P. D’Agostino

**Affiliations:** 1 Biology Department, Boston College, Chestnut Hill, MA 02467 USA; 2 George Washington University Medical Center Washington DC, and Aegis Medical & Research Associates Annapolis, Maryland, USA; 3 Department of Neurosurgery, University of Pittsburgh Medical Center, Suite 5C, 200 Lothrop St, Pittsburgh, PA USA; 4 Department of Molecular Pharmacology and Physiology, University of South Florida, Tampa, Florida USA

**Keywords:** Glucose, Glutamine, Mitochondria, KETONE bodies, Diet, Warburg effect, Cancer metabolism, Glutaminolysis, Hyperbaric oxygen

## Abstract

**Background:**

A shift from respiration to fermentation is a common metabolic hallmark of cancer cells. As a result, glucose and glutamine become the prime fuels for driving the dysregulated growth of tumors. The simultaneous occurrence of “Press-Pulse” disturbances was considered the mechanism responsible for reduction of organic populations during prior evolutionary epochs. Press disturbances produce chronic stress, while pulse disturbances produce acute stress on populations. It was only when both disturbances coincide that population reduction occurred.

**Methods:**

This general concept can be applied to the management of cancer by creating chronic metabolic stresses on tumor cell energy metabolism (press disturbance) that are coupled to a series of acute metabolic stressors that restrict glucose and glutamine availability while also stimulating cancer-specific oxidative stress (pulse disturbances). The elevation of non-fermentable ketone bodies protect normal cells from energy stress while further enhancing energy stress in tumor cells that lack the metabolic flexibility to use ketones as an efficient energy source. Mitochondrial abnormalities and genetic mutations make tumor cells vulnerable metabolic stress.

**Results:**

The press-pulse therapeutic strategy for cancer management is illustrated with calorie restricted ketogenic diets (KD-R) used together with drugs and procedures that create both chronic and intermittent acute stress on tumor cell energy metabolism, while protecting and enhancing the energy metabolism of normal cells.

**Conclusions:**

Optimization of dosing, timing, and scheduling of the press-pulse therapeutic strategy will facilitate the eradication of tumor cells with minimal patient toxicity. This therapeutic strategy can be used as a framework for the design of clinical trials for the non-toxic management of most cancers.

## Background

According to the paleobiologists, Arens and West, the simultaneous occurrence of “Press-Pulse” disturbances was considered the mechanism responsible for the extinction of organic populations during prior evolutionary epochs [1]. A “press” disturbance was considered a chronic environmental stress on all organisms in an ecological community. The press disturbance promoted extinction through habitat loss, reduced reproduction, and restriction of range and resources. Press disturbances would force a biological community into a new equilibrium where previously important species become non-viable. A press disturbance would shift the adaptive landscape to favor the fittest species while eliminating the weakest species. In contrast to the press disturbances, “pulse” disturbances were considered acute events that disrupted biological communities to produce high mortality [1]. Through extensive mortality in the immediate aftermath of the event, a pulse disturbance could cause extinction. However, survival of some species could occur following a pulse disturbance, as the physical and biotic environments would eventually recover to their pre-disturbance equilibria [1]. It was only when both the press and the pulse disturbances coincided that mass extinction of species, without recovery, was possible. We describe how a modification of the press-pulse concept can be adopted as a therapeutic strategy for the possible eradication of tumor cells. The press-pulse concept should be best considered in light of current views on the origin of cancer.

### The origin of cancer

Cancer is a systemic disease involving multiple time- and space-dependent changes in the health status of cells and tissues that ultimately lead to malignant tumors [2]. Neoplasia involving dysregulated cell growth is the biological endpoint of the disease [3, 4]. Tumor cell invasion into surrounding tissues and their spread (metastasis) to distant organs is the primary cause of morbidity and mortality of most cancer patients [5–9]. Data from the American Cancer Society show that the rate of increase in cancer deaths/year (3.4%) was two-fold greater than the rate of increase in new cases/year (1.7%) from 2013 to 2017 [10, 11]. Indeed, cancer is predicted to overtake heart disease as the leading cause of death in Western societies. The failure to clearly define the origin of cancer is responsible in large part for the failure to significantly reduce the cancer death rate from treatments and in developing cancer prevention strategies [12].

Cancer is generally considered a genetic disease where random somatic mutations underlie the origin and progression of the disease [4, 13–16]. This general view is now under serious reconsideration in light of major inconsistencies with the gene theory [2, 3, 12, 14, 17–24]. Emerging evidence from the cancer genome projects shows that most malignant tumors are remarkably heterogeneous [2, 15, 16, 25–27]. This degree of heterogeneity will confound attempts to exploit genomic defects for effective therapies. Moreover, the majority of genetic mutations are considered downstream epiphenomena of dysregulated energy metabolism [2, 20, 28]. In contrast to the extensive genetic heterogeneity seen in tumors, most if not all neoplastic cells within tumors share the common metabolic malady of aerobic fermentation that arises ultimately from dysregulated oxidative phosphorylation [2, 17, 29–33]. In light of these findings, cancer can also be recognized as a metabolic disease.

## Methods

### Aerobic fermentation: a common metabolic malady of tumor cells

Most cells of the body oxidize glucose to CO_2_ and water for energy production. Before entering the mitochondria for complete oxidation, glucose is first split into two molecules of pyruvate through the Embden–Meyerhof–Parnas glycolytic pathway in the cytosol. As most cells are bathed in oxygen, the production of pyruvate occurs through aerobic glycolysis [34]. Under hypoxia, however, much of the pyruvate is reduced to lactic acid in order to maintain cell ATP production. Aerobic fermentation, on the other hand, involves the production of lactic acid under normoxic conditions. As the Pasteur effect should reduce lactic acid fermentation under normoxia, persistent lactic acid production in the presence of adequate oxygen is indicative of abnormal respiration [35]. Otto Warburg first proposed that all cancers arise from damage to cellular respiration. As a result, cancer cells increase their capacity to produce lactic acid even in the presence of oxygen in order to compensate for their insufficient respiration [31, 36].

Although Warburg’s hypothesis on the origin of cancer has created confusion and controversy [37–40], his hypothesis has never been disproved. The Crabtree effect and the high oxygen consumption rate seen in some tumor cells have confused the picture of defective oxidative phosphorylation in tumor cells. The Crabtree effect is an artifact of the in vitro environment and involves the glucose-induced suppression of respiration with a corresponding elevation of lactic acid production even under hyperoxic (pO^2^ = 120–160 mmHg) conditions associated with cell culture, [41, 42]. Also, the oxygen consumption seen in tumor cells is not always linked to ATP production through oxidative phosphorylation and cannot therefore be used alone as evidence of normal respiration [29, 43–48]. It can be difficult to accurately measure mitochondrial respiratory function in cultured cells unless appropriate controls are used, as the in vitro environment can alter mitochondrial function [41, 49]. These issues have confounded the interpretation of Warburg’s findings despite his attempts to clarify the issues [32, 48, 50]. Nevertheless, the Warburg theory of insufficient aerobic respiration remains as the most credible explanation for the origin of tumor cells [2, 37, 51–57].

The main points of Warburg’s theory are; 1) insufficient respiration is the predisposing initiator of tumorigenesis and ultimately cancer, 2) energy through glycolysis gradually compensates for insufficient energy through respiration, 3) cancer cells continue to produce lactic acid in the presence of oxygen, and 4) respiratory insufficiency eventually becomes irreversible [2, 31, 32, 36, 58, 59]. Warburg referred to the phenomenon of enhanced glycolysis in cancer cells as “aerobic fermentation” to highlight the abnormal production of lactic acid in the presence of oxygen [31, 32, 36, 58, 59]. Efraim Racker coined the term “Warburg effect”, which refers to the aerobic glycolysis that occurs in cancer cells [60]. Although Warburg insisted that aerobic glycolysis confuses the issue of insufficient respiration as the origin of cancer [31, 32], some in the cancer metabolism field have persisted in thinking that aerobic glycolysis (Warburg effect) is a central issue in cancer metabolism [39, 61]. Warburg clearly demonstrated that aerobic fermentation (aerobic glycolysis) is an effect, and not the cause, of insufficient respiration [36]. Hence, the targeting of fermentable fuels becomes of prime importance for cancer management.

Substantial evidence exists showing that many cancers avidly consume glucose and produce lactic acid [62–67]. The diagnostic procedure of ^18^F-deoxyglucose positron emission tomography (FDG-PET) is considered evidence for the elevated use of glucose by some tumors [66]. Elevated glucose consumption would be expected for any glucose-dependent cell with quantitative or qualitative abnormalities in mitochondria, as enhanced fermentation would be needed to compensate for the insufficient respiration [43, 68]. Indeed, all tumor cells that have been examined to date contain abnormalities in the content or composition of cardiolipin, the signature lipid of the inner mitochondrial membrane that regulates oxidative phosphorylation [69–74]. Mammalian cells containing abnormalities in cardiolipin cannot respire effectively and will therefore need to increase energy production through fermentation reactions [41, 70, 73, 75–78]. This fact cannot be overemphasized considering arguments that tumor cells can have normal respiration [39, 61, 79]. The expression of immature cardiolipin linked to reduced Complex I activity in the inner mitochondrial membrane of tumorigenic and non-tumorigenic cells suggests that many proliferative cells grown in culture obtain energy through fermentation rather than through oxidative phosphorylation despite the appearance of normal oxygen consumption [41, 43]. The cardiolipin abnormalities found in tumor cells provide direct support for Warburg’s central theory. In addition to cardiolipin abnormalities, Pedersen also showed that some degree of abnormality could be found in the number, structure, or function of tumor cell mitochondria providing further support for Warburg’s theory [68]. The evidence supporting Warburg’s original theory comes from a broad range of cancers and is now overwhelming [2, 36, 53, 80–85]. Hence, respiratory insufficiency, arising from any number mitochondrial defects, can contribute to the fermentation metabolism seen in tumor cells.

Although the abnormal energy metabolism and mitochondrial abnormalities seen in most cancers could arise in part through oncogenic modulation of metabolism [4, 39, 86], the data from the nuclear and mitochondrial transfer experiments suggest that oncogene changes are effects, rather than causes, of tumorigenesis [2, 14, 24, 87, 88]. Normal mitochondria can suppress tumorigenesis, whereas abnormal mitochondria can enhance tumorigenesis [14, 87]. The results from these experiments must be viewed together, as results from any given single experiment are not capable of overturning the gene theory [14]. Recent advances in CRISPR/Cas9 technology might help to generate nuclei with changes in specific tumor-associated genes to further evaluate the influence of gene mutations and mitochondrial function on tumorigenesis. The acquisition of dysfunctional mitochondria in macrophages through fusion hybridization with non-metastatic tumor cells provides a compelling argument for the origin of those cancer cells that become metastatic [5, 89–91]. We recently showed how all of the Hanahan & Weinberg hallmarks of cancer, including the genomic mutations, could be linked either directly or indirectly to mitochondrial dysfunction [2, 56, 92].

### Amino acid fermentation could also drive cancer metabolism

As the result of insufficient aerobic respiration, cancer cells must rely primarily on fermentation metabolism to maintain energy balance and viability. Besides substrate level phosphorylation in the cytoplasm through lactic acid fermentation, TCA cycle substrate level phosphorylation can also produce significant amounts ATP [93–98]. In addition to glucose, cancer cells also rely heavily on glutamine for growth and survival [99–102]. Glutamine is anapleurotic and can be rapidly metabolized to glutamate and then to α-ketoglutarate for entry into the TCA cycle. In addition to serving as a carbon/nitrogen source for tumor cell growth, glutamine also plays a role in cancer cell survival and growth through enzymatic release of ammonia into the microenvironment [103]. The TCA cycle succinate thiokinase reaction could generate the majority of cellular ATP through substrate level phosphorylation under hypoxia or in tumor cells with defective oxidative phosphorylation [78]. Mitochondrial ATP production through TCA cycle substrate level phosphorylation, using glutamine as a substrate, could give the appearance that mitochondrial energy metabolism is normal in some cancer cells especially in combination with oxygen consumption and CO_2_ production. Although Warburg did not address the role of TCA cycle substrate level phosphorylation in his original work [31, 36], an increase in TCA cycle substrate level phosphorylation would be expected in cells with OxPhos deficiencies, just as lactic acid fermentation is expected in cells with this deficiency. Further studies will be needed to substantiate the role of glutamine fermentation in cancer cells.

Glucose and glutamine act synergistically for driving rapid tumor cell growth. Glutamine metabolism can produce ATP from the TCA cycle under aerobic conditions. Glutamine is also a nitrogen donor for nucleotide biosynthesis and can serve as precursor for lipid synthesis under hypoxic conditions [104, 105]. We also found that only minor amounts of glutamine are metabolized to lactic acid under either normoxia or hypoxia in the VM-M3 invasive glioblastoma cells consistent with findings in other tumor cells [105–107]. We suggest that the metabolism of glucose and glutamine for energy will depend on the physiological state of the tumor microenvironment, and will be of greater significance in tumors with an aggressive Warburg phenotype. We found that glutamine targeting can be effective in managing systemic metastatic cancer in the VM/Dk mice [108].

Amino acid fermentation can generate energy through TCA cycle substrate level phosphorylation under hypoxic conditions [94, 96, 97, 109, 110]. Succinate is a waste product of amino acid fermentation that can enhance inflammation as well as inhibit a family of prolyl hydroxylases, which facilitate Hif-1α degradation through the von Hippel–Lindau (VHL) gene product [111–113]. Through its action on several glycolytic pathways, Hif-1α stabilization enhances aerobic fermentation [114–116]. It can be difficult to determine, however, the degree to which mitochondrial ATP production in tumor cells arises from coupled respiration or from TCA cycle substrate level phosphorylation [94, 98].

**Fig. 1:**
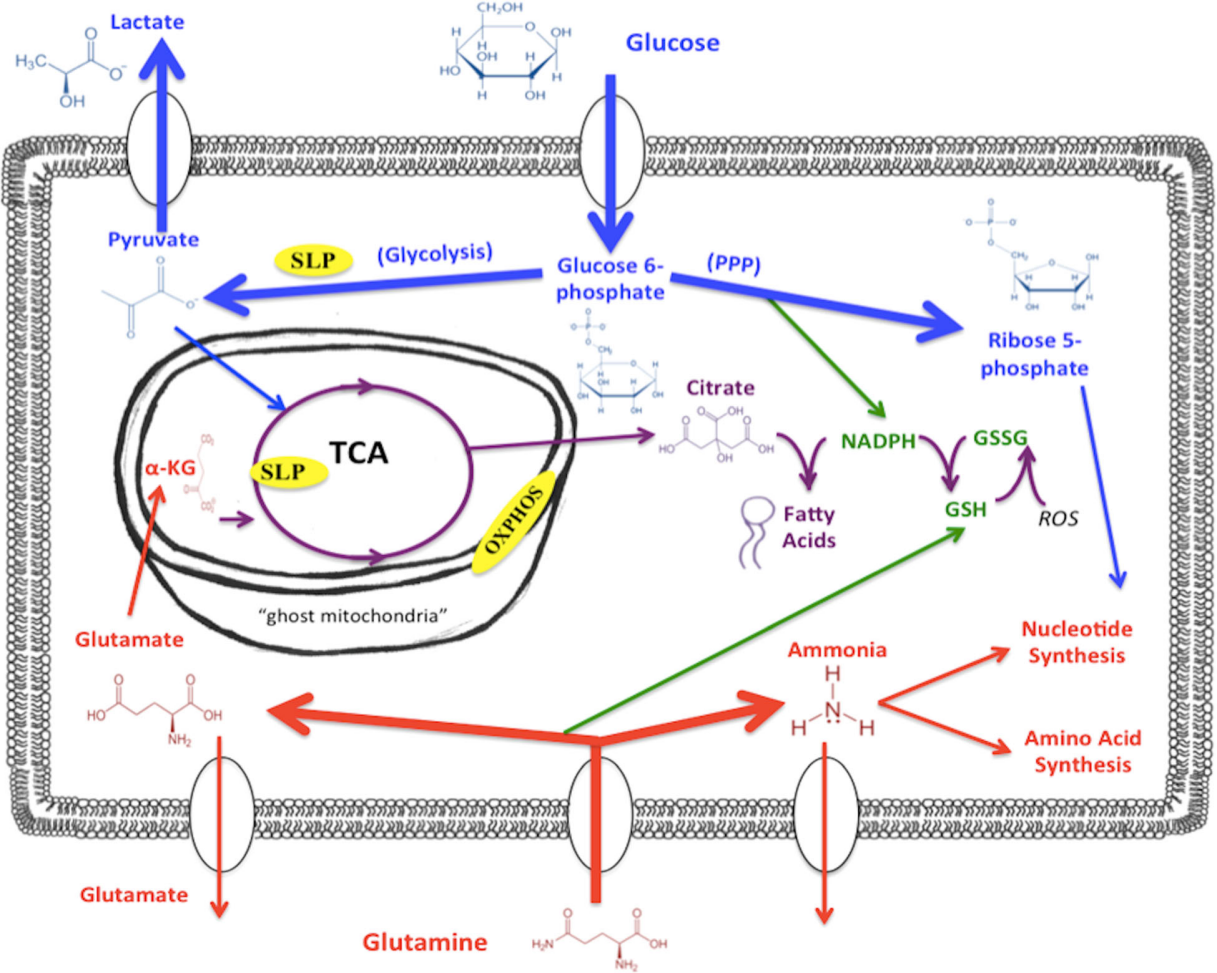
Targeting Glucose and Glutamine for the Metabolic Management of Cancer. Cancer cells are largely dependent on glucose and glutamine for survival and growth. Energy through fermentation metabolism (substrate level phosphorylations, SLP) in glycolysis and the tricarboxylic acid cycle (TCA) will compensate for reduced energy through oxidative phosphorylation (OxPhos) that occurs in tumor cells. The yellow ovals indicate the three source of cellular ATP production. Glucose carbons can be used for both the glycolytic and pentose phosphate (PPP) pathways to supply ATP and precursors for lipid and nucleotide synthesis, as well as for glutathione production. Glutamine provides its amide nitrogen for ammonia and nucleotide synthesis. The glutamine-derived glutamate provides anapleurotic carbons to the TCA cycle through α-KG for protein synthesis while also providing ATP through TCA cycle SLP. TCA cycle substrate level phosphorylation through the succinate thiokinase reaction can generate significant cellular ATP under hypoxia especially in tumor cells with defective respiration [78]. The glutamine-derived glutamate is also used for glutathione production that protects tumor cells from oxidative stress. Glucose and glutamine targeting will thus make cancer cells vulnerable to oxidative stress therapies. The simultaneous targeting of glucose and glutamine through the press-pulse therapeutic strategy will starve tumor cells of energy production while blocking their ability to synthesize proteins, lipids, and nucleotides. Glucose and glutamine can also be generated internally through the lysosomal digestion of phagocytosed glycoconjugates and proteins (see text). An elevation of non-fermentable ketone bodies through, calorie restriction, ketogenic diets, or supplementation will provide normal cells with an alternative energy source to glucose while also protecting them from oxidative stress. Ghost mitochondria are those containing little or no inner mitochondrial membranes (cristae), which are essential for normal OxPhos function [67, 282, 283]

Several byproducts of amino acid fermentation can also accumulate in the tumor microenvironment including acetate, glutamate, alanine, succinate, and ammonia. Although acetate has been considered a potential fuel for supporting tumor cell growth [117, 118], acetate levels are generally low in the circulation [119]. Jaworski et al. recently provided a comprehensive discussion on the potential role of acetate in tumor metabolism [120]. It should be recognized that with the exception of glucose and glutamine, none of the other potential fuels needed for tumor cell fermentation would likely be available in sufficient quantities to drive robust tumor cell growth. As many amino acids are synthesized from glucose and glutamine, targeting glucose and glutamine will deprive the microenvironment of fermentable fuels. Hence, the restriction of glucose and glutamine becomes of prime importance for targeting tumor cell growth and survival. The role of glucose and glutamine in driving tumor cells energy metabolism is shown in Fig. 1.

### Tumor cell energy metabolites from cannibalism and phagocytosis

Emerging evidence indicates that macrophages, or their fusion hybridization with neoplastic stem cells, are the origin of metastatic cancer cells [5, 89, 121–124]. Radiation therapy can enhance fusion hybridization that could increase risk for invasive and metastatic tumor cells [91, 125]. Cannibalism and phagocytosis of cellular debris are well known characteristics of macrophages and of myeloid cancer cells with macrophage properties [121, 126–131]. Shelton showed that glioblastoma cells with myeloid properties could survive in Matrigel (extracellular matrix material) in the absence of added glucose and glutamine [132]. The gradual accumulation of lactate in the media suggested that the glioblastoma cells survived through lysosomal digestion and aerobic fermentation of glycoconjugates present in the Matrigel. Glioblastoma cell death occurred immediately following the addition of chloroquine, which neutralizes lysosomal acidity and digestion [132]. Shelton’s findings are consistent with the more recent findings of Kamphorst et al. in showing that pancreatic ductal adenocarcinoma cells could obtain glutamine under nutrient poor conditions through lysosomal digestion of extracellular proteins [133]. It will therefore become necessary to also target lysosomal digestion, under reduced glucose and glutamine conditions, to effectively manage those invasive and metastatic cancers that express cannibalism and phagocytosis.

### Genome integrity and energy metabolism

Emerging evidence indicates that the function of DNA repair enzymes and the integrity of the nuclear genome are dependent to a large extent on the energy derived from normal respiration [134–142]. Previous studies in yeast and mammalian cells show that disruption of aerobic respiration can cause mutations (loss of heterozygosity, chromosome instability, and epigenetic modifications etc.) in the nuclear genome [28, 141, 143, 144]. A protracted reliance on fermentation causes oxidative stress leading to the production of reactive oxygen species (ROS) mostly through the mitochondrial coenzyme Q couple [145]. In addition to their role in oncogenic signaling, excess ROS production damages mitochondrial function, and can be both carcinogenic and mutagenic [146, 147]. The somatic mutations and genomic instability seen in tumor cells thus arise from a protracted reliance on fermentation energy metabolism and a disruption of redox balance through excess oxidative stress.

We recently discussed how a transition from respiration to fermentation could account for Szent-Gyorgi’s “Oncogenic Paradox”, i.e., the process by which various provocative agents (radiation, inflammation, hypoxia, carcinogenic chemicals, age, germline mutations, etc.) could produce cancer through a common pathological mechanism [2, 148]. Mukherjee and Cairns also struggled to explain the oncogenic paradox [149, 150]. All of these provocative cancer-causing agents damage respiration thus forcing the cells to rely more heavily on energy generated through fermentation for survival. According to the mitochondrial metabolic theory of cancer, the large genomic heterogeneity seen in tumor cells arises as a consequence, rather than as a cause, of mitochondrial dysfunction [2, 14, 28]. A therapeutic strategy targeting the metabolic abnormality common to most tumor cells should therefore be more effective in managing cancer than would a strategy targeting genetic mutations that vary widely between tumors of the same histological grade and even within the same tumor.

### Human evolution and adaptive versatility

Rick Potts, a paleoanthropologist at the Smithsonian Institution, suggested that the evolutionary success of our species has been due largely to the germline inheritance of traits that bestowed adaptive versatility [151–153]. Adaptability was defined in terms of, 1) the ability of an organism to persist through major environmental shifts, 2) to spread to new habitats, and 3) to respond in novel ways to its surroundings [153]. These characteristics were honed over millions of years and enabled humans to adapt rapidly to abrupt changes in the physical environment including changes in moisture, temperature, food resources etc. Adaptability to abrupt environmental change is a property of the genome, which was selected for in order to ensure survival under environmental extremes [65, 154].

Potts’ hypothesis is an extension of Darwin’s original theory (Chapter IV, Natural Selection) and can be applied to the individual cells of the organism, which exist as an integrated society of cells [65, 154]. The success in dealing with environmental stress and disease is therefore dependent on the integrated action of all cells in the organism. Further, this integrated action depends on the flexibility of each cell’s genome, which responds to both internal and external signals according to the needs of the organism. More specifically, only those cells possessing flexibility in nutrient utilization will be able to survive under nutrient stress. Environmental forcing has therefore selected those genomes most capable of adapting to change in order to maintain metabolic homeostasis [65, 152, 153, 155]. This concept was first discussed in relationship to the management of brain cancer [65].

The widely held notion that tumor cells have a growth advantage and are more fit than normal cells are in contrast to Darwin’s theory of evolution and also to Potts’ theory of adaptive versatility [65, 153, 154]. It is difficult to conceive how a random accumulation of somatic mutations could enhance the adaptability and fitness of cancer cells. It is important to recognize that mutations in *p53*, *K-Ras*, and *Raf* impact negatively on mitochondrial energy efficiency thus making cells with these mutations less metabolically flexible than normal cells [28, 44, 53, 135, 156–159]. Indeed activating mutations in *K-Ras* target mitochondria, thus enhancing glycolysis [53, 160]. Enhanced glycolysis will make tumor cells appear more metabolically fit than normal cells in hypoxic environments [161, 162]. Most normal cells, however, cannot grow in hypoxia and will often die in hypoxic environments due to respiratory failure. Tumor cells are more fit than normal cells to survive in the hypoxic niche of the tumor microenvironment. Hypoxic adaptation of tumor cells allows for them to avoid apoptosis due to their metabolic reprograming following a gradual loss of respiratory function [31, 32, 162, 163]. The high rates of tumor cell glycolysis and glutaminolysis will also make them resistant to apoptosis, ROS, and chemotherapy drugs [163]. Despite having high levels of ROS, glutamate-derived from glutamine contributes to glutathione production that can protect tumor cells from ROS [164]. As long as the tumor cells have access to the metabolic fuels needed for glycolysis and TCA cycle substrate level phosphorylation (glucose and glutamine, respectively) they will give the appearance of having a growth advantage over most normal cells [2]. According to Darwin and Potts, mutations that bestow a selective advantage are those that will enhance survival under environmental stress. If the multiple pathogenic point mutations, chromosomal rearrangements, and mitochondrial abnormalities confer a fitness or survival advantage to tumor cells, then survival under environmental stress and nutrient deprivation should be better in tumor cells than in normal cells [165]. This is not what actually happens, however, when the hypothesis is tested.

For example, when mice or people with tumors are placed under energy stress using dietary energy reduction (glucose restriction), many tumor cells die while normal cells survive. Indeed, the health and vitality of the normal cells improves with time under dietary energy reduction while hyper-glycolytic tumor cells undergo energetic crisis triggering apoptotic death [166, 167]. Support for this contention comes from studies of treating brain tumors with dietary energy stress [114, 168–174]. It is clear that adaptability to environmental stress is greater in normal cells than in tumor cells, as normal cells can transition from the metabolism of glucose to the metabolism of ketone bodies when glucose becomes limiting. Mitochondrial oxidative phosphorylation is less robust in tumor cells than in normal cells while glucose utilization through lactic acid fermentation is greater in tumor cells than in normal cells. Targeting glucose availability will therefore cause greater death in the tumor cells than in the normal cells. Mitochondrial respiratory chain defects will prevent tumor cells from using ketone bodies for energy [145]. Consequently, glycolysis-dependent tumor cells are less adaptable to metabolic stress than are the normal cells. This vulnerability can be exploited for targeting tumor cell energy metabolism [160, 163].

It is also possible that therapeutic energy stress could restore the microenvironment thus reversing abnormal energy metabolism and growth behavior in tumor cells not containing genetic mutations [19, 175]. In contrast to dietary energy reduction, radiation and toxic drugs can damage the microenvironment and transform normal cells into tumor cells while also creating tumor cells that become highly resistant to drugs and radiation. Drug-resistant tumor cells arise in large part from the damage to respiration in bystander pre-cancerous cells. These cells are often those that eventually become heavily dependent on fermentation for energy.

The greater adaptability of normal cells than tumor cells to energy stress is predicted based on the theories of Darwin and Potts [154]. Metabolic flexibility allows the organism to respond in a coordinated way to environmental stress and limited substrate availability. Energy stress will force all normal cells to work together for the survival of the organism [154]. Pathogenic mutations and genomic instability will reduce adaptability and metabolic flexibility under energy stress. The greater the genomic instability in tumor cells, the less will be their adaptability to stress. This concept is similar to that of Nowell’s except in viewing genomic instability as a liability rather than as an advantage to progression [154, 176]. Because energy generated through substrate level phosphorylation is greater in tumor cells than in normal cells, tumor cells are more dependent than normal cells on the availability of fermentable fuels (glucose and glutamine) [94]. With few exceptions, most normal cells shift energy metabolism from glucose to ketone bodies and fats when placed under energy stress from glucose deprivation, insulin deficiency, and prolonged fasting. This shift is the result of adaptive versatility and genomic stability, which is lacking in the tumor cells but is prominent in cells and tissues with robust mitochondrial function.

Tumor cells will have difficulty surviving and growing, regardless of their complement of genomic changes, if fermentable fuels become restricted in the microenvironment. Ketone bodies and fats are non-fermentable fuels [177]. Tumor cells have difficulty using ketone bodies and fats for fuel when glucose is reduced [57, 178–180]. Although some tumor cells might appear to oxidize ketone bodies by the presence of ketolytic enzymes [181], it is not clear if ketone bodies and fats can provide sufficient energy for cell viability in the absence of glucose and glutamine. The studies in immunocompetent syngeneic mice and xenografts with brain tumors are proof of concept that tumor cells are less adaptable than normal cells when placed under energy stress [114, 170, 171, 182–184]. Apoptosis under energy stress is greater in tumor cells than in normal cells [170]. The multiple genetic defects in tumor cells will reduce genomic flexibility thus increasing the likelihood of cell death under environmental stress that would lower glucose and elevate ketone bodies. Regardless of when or how genomic defects become involved in the initiation or progression of tumors, these defects can be exploited for tumor management or resolution [12].

## Results

### Press-pulse: a therapeutic strategy for the gradual elimination of cancer cells

Mark Vincent suggested how a Press-Pulse strategy could be used to target tumor cells [185]. We have now expanded this concept to show how a press-pulse therapeutic strategy can be used for the non-toxic management and possible resolution of cancer. A calorie restricted ketogenic diet or dietary energy reduction creates chronic metabolic stress in the body. This energy stress acts as a press disturbance; the effects of which would be greater in the tumor cells than in the normal cells due to their dependency on fermentation energy metabolism, mitogens, anabolic signaling (IGF-1, mTOR, etc.), elevated redox stress, and mutational load. Drugs that target availability of glucose and glutamine would act as pulse disturbances in causing an acute reduction of these tumor-dependent fuels [186]. Hyperbaric oxygen therapy can also be considered another pulse disturbance in elevating ROS to a greater degree in tumor cells than in normal cells, thus promoting cancer cell death through redox stress [40]. Normal cells readily transition to ketone body metabolism for protection against ROS damage and oxidative stress. The goal therefore is to produce a therapeutic strategy that can more effectively manage cancer than can the toxic cancer therapies currently used in most standards of care. The following examples illustrate the potential of press-pulse therapeutic strategies for cancer management.

### Calorie restriction and restricted Ketogenic diets: a press disturbance

Calorie restriction, water-only fasting, and restricted ketogenic diets reduce circulating glucose and insulin levels while elevating circulating levels of ketone bodies. Ketogenic diets (KDs) are low carbohydrate-high fat diets that are widely used to reduce refractory epileptic seizures in children [187, 188]. The KD can more effectively reduce glucose and elevate blood ketone bodies than can CR alone making the KD potentially more therapeutic against tumors than CR [174, 189, 190]. The protein and fat composition of the KD differs from that of Atkins-type diets in having comparatively less protein and more fat than the Atkins diets. This is important as several amino acids found in proteins can be deaminated to form pyruvate, which can then be metabolized to form glucose through gluconeogenesis [191]. Campbell showed that tumor growth in rats is greater under high protein (>20%) than under low protein content (<10%) in the diet [192]. Protein amino acids can be metabolized to glucose through the Cori cycle. The fats in KDs used clinically also contain more medium chain triglycerides than do Atkins diets. Consequently, blood glucose levels will be lower and ketone body levels will be higher with KDs than with Atkins-type diets. Calorie restriction, fasting, and restricted KDs are anti-angiogenic, anti-inflammatory, and pro-apoptotic and thus can target and eliminate tumor cells through multiple mechanisms [114, 166, 171, 174, 182, 193, 194]. Ketogenic diets can also spare muscle protein, enhance immunity, and delay cancer cachexia, which is a major problem in managing metastatic cancer [195–198].

The therapeutic effects of KDs used alone or in combination with other therapies have been documented in preclinical studies for several cancer models including neuroblastoma [199, 200], lung cancer [201], prostate cancer [202, 203], breast and ovarian cancers [204, 205], head & neck cancers [204], colon cancer [206], and pancreatic cancer [198]. These preclinical studies are also motivating case reports and pilot studies in humans with brain cancer and other cancers [169, 181, 207–214]. It is clear from these studies and other studies in children and adults with cancer that KDs are generally safe and well tolerated [168, 212, 213, 215–217], These observations are also consistent with decades of research obtained from evaluation of children treated with KDs for epilepsy management [218]. Information on ketogenic diets can be obtained from the Charlie Foundation web site (https://www.charliefoundation.org).

We recently developed the Glucose/Ketone Index calculator (GKIC) to assess the potential therapeutic effects of various low-carbohydrate and KDs for brain cancer management [189]. The GKIC is a simple tool that measures the ratio of blood glucose to blood ketones and can help monitor the efficacy of metabolic therapy in preclinical animal models and in clinical trials for malignant brain cancer or for any cancer that expresses aerobic fermentation. GKI values of 1.0 or below are considered therapeutic, though therapeutic benefit appears to be associated more with elevated ketone bodies and suppression of insulin than with reduced glucose [190, 215]. However, the elevation of ketone body levels is generally greater when blood glucose levels are lower than when glucose levels are higher [174, 219, 220]. The GKI can therefore serve as a biomarker to assess the therapeutic efficacy of various diets in a broad range of cancers.

Reduced glucose availability and suppression of insulin signaling will produce chronic energy stress on those tumor cells that depend primarily on glucose for growth and survival. It is important to remember that insulin drives glycolysis through stimulation of the pyruvate dehydrogenase complex [221, 222]. Reduced levels of glucose will also reduce substrates for both the glycolytic and the pentose phosphate pathways thus reducing cellular energy, and the synthesis of glutathione and nucleotide precursors (Fig. 1).

The water-soluble ketone bodies (D-β-hydroxybutyrate and acetoacetate) are produced largely in the liver from adipocyte-derived fatty acids and ketogenic dietary fat. Ketone bodies bypass glycolysis and directly enter the mitochondria for metabolism to acetyl-CoA [223]. In contrast to fatty acid metabolism, which generates both NADH and FADH_2_, ketone body metabolism generates only NADH [145]. Moreover, ketone body metabolism does not induce mitochondrial uncoupling in contrast to metabolism of saturated fatty acids [145]. The metabolism of D-β-hydroxybutyrate in normal cells will therefore increase the redox span between Complexes I and III, thus increasing the delta G’ of ATP hydrolysis while, at the same time, reducing ROS formation through the Complex II coenzyme Q couple [224, 225]. Due to mitochondrial defects, tumor cells cannot exploit the therapeutic benefits of burning ketone bodies as normal cells would. Indeed, racemic mixtures of D-/L-ketone bodies can be toxic to tumor cells under both low and high glucose conditions [57, 190]. Fine et al. found that uncoupling protein 2 is overexpressed in tumor cells, but not in normal control cells [226]. This finding provides a plausible molecular mechanism by which ketone bodies spare normal cells but suppresses growth in cancer lines.

In contrast to D-β-hydroxybutyrate, L-β-hydroxybutyrate is beta-oxidized thus producing both NADH and FADH_2_. FADH_2_ will deliver electrons to Complex III, which can increase the semiquinone of Q, the half-reduced form. The Q semiquinone will react with molecular oxygen to form the superoxide O_2_^.-^ free radical [145]. Therapeutic ketosis with racemic ketone esters can also make it feasible to safely sustain hypoglycemia for inducing metabolic stress on cancer cells [227]. Hence, mixtures of L- and D-ketone esters have the potential to both enhance oxidative stress in tumor cells while reducing oxidative stress in normal cells, respectively [145, 228]. There is also evidence showing that ketone bodies can inhibit histone deacetylases (HDAC) [229]. HDAC inhibitors play a role in targeting the cancer epigenome [230]. Deregulated inflammation is also considered to be one of the hallmarks of cancer. Therapeutic ketosis reduces circulating inflammatory markers, and ketones directly inhibit the NLRP3 inflammasome, an important pro-inflammatory pathway linked to carcinogenesis and an important target for cancer treatment response [231]. There are no adverse side effects of short-term therapeutic ketosis, but relatively mild adverse effects have been noted in some children with epilepsy after long-term use of ketogenic diets including constipation, kidney stones, electrolyte imbalances, and bone fracture [218]. These adverse effects were easily managed with various supplements and pale in comparison to the adverse effects produced from current standards of care [232]. In general, there are no currently known cancer drugs that embody the therapeutic properties of ketone bodies.

### Psychological stress reduction: a press disturbance

Chronic psychological stress is known to promote tumorigenesis through elevations of blood glucose, glucocorticoids, catecholamines, and insulin-like growth factor (IGF-1) [233, 234]. In addition to calorie-restricted ketogenic diets, psychological stress management involving exercise, yoga, music etc. also act as press disturbances that can help reduce fatigue, depression, and anxiety in cancer patients and in animal models [235–238]. Ketone supplementation has also been shown to reduce anxiety behavior in animal models [239]. The mechanism of action of psychological stress management for cancer control would largely involve reductions in blood glucose levels that contribute to tumor growth.

### Restricted ketogenic diet used with 2-Deoxyglucose

Calorie restriction or therapeutic fasting is anti-angiogenic, anti-inflammatory, and pro-apoptotic, and thus targets multiple cancer hallmarks [114, 166, 167, 170, 171, 182, 240–243]. This physiological state also enhances the efficacy of chemotherapy and radiation therapy, while reducing the side effects [244–246]. Indeed, lower dosages of chemotherapeutic drugs can be used when administered together with calorie restriction or restricted ketogenic diets (KD-R). We showed a synergistic interaction between a KD-R and the glycolysis inhibitor 2-deoxyglucose (2-DG) for the metabolic management of the syngeneic CT-2A malignant mouse glioma [247]. It was interesting to find that 2-DG (25 mg/kg) had no therapeutic effect on CT-2A tumor growth when administered alone to mice on a standard high carbohydrate diet, but had a powerful therapeutic effect when administered with a KD-R. Indeed, this relatively low dose of 2-DG became somewhat toxic when used with the KD suggesting that lower dosing of some tumor-targeting drugs could also be effective when administered with KD-R. Besides 2-DG, a range of other glycolysis inhibitors might also produce similar therapeutic effects when combined with the KD-R including 3-bromopyruvate, oxaloacetate, and lonidamine [58, 186, 248–250]. In the example here the KD-R is the press making cancer cells selectively vulnerable to death and the 2-DG is the pulse, which could be used intermittently or cycled to avoid toxicity.

### Ketogenic diet used with radiation therapy

Adrienne Scheck and colleagues showed that the therapeutic efficacy of radiotherapy against the orthotopically grown GL261 mouse glioma could be greatly enhanced when combined with a commercially available ketogenic diet [183]. Mice fed the KetoCal ketogenic diet had elevated levels of β-hydroxybutyrate and an increased median survival of approximately 5 days relative to animals maintained on a high-carbohydrate standard diet alone. A synergistic interaction of the KD diet plus radiation was seen, as no bioluminescent signal was detected in 9 of 11 that received the combined treatment. Furthermore, no signs of tumor recurrence were seen for over 200 days when the treated mice were switched to the SD 101 days after tumor implantation. These findings suggest tumor resolution in some of the mice treated with the combined therapy. In this example, the KD is the press and radiotherapy is the pulse. It is important to recognize, however, that the radiotherapy used in glioma patients can damage the respiration of normal cells and increase availability of glutamine in the microenvironment, which can increase risk of tumor recurrence especially when used together with the steroid drug dexamethasone [31, 251–253].

### A Ketogenic diet used with hyperbaric oxygen therapy

Poff and colleagues demonstrated that hyperbaric oxygen therapy (HBOT) enhanced the ability of the KD to reduce tumor growth and metastasis [40]. Evidence in animal models and in humans suggests that HBOT may have a modest anti-cancer effect when used alone [254], but appears most efficacious when it is used in combination with standard care. Indeed, HBOT has proven effective when used prior to radiation therapy for GBM [255]. The mechanism of HBOT in tumor management is not yet clear, but saturating the tumor with oxygen could reverse hypoxia and suppresses growth [254, 256] HBOT also increases oxidative stress and membrane lipid peroxidation of GBM cells in vitro [257]. The effects of the KD and HBOT can be enhanced with administration of exogenous ketones, which further suppressed tumor growth and metastasis [190]. Besides HBOT, intravenous vitamin C and dichloroacetate (DCA) can also be used with the KD to selectively increase oxidative stress in tumor cells [258, 259]. Recent evidence also shows that ketone supplementation may enhance or preserve overall physical and mental health [260, 261], which are often compromised due to disease progression and standard of care therapies. Under these conditions the KD with exogenous ketones serve as the press, while HBOT serves as the pulse. Although HBOT and radiotherapy kill tumor cells through oxidative stress, HBOT is less toxic to normal cells than is radiotherapy.

### Calorie restriction used with glutamine targeting for metastatic cancer

Some tumors use glucose as a prime fuel for growth, whereas other tumors use glutamine as a prime fuel [102, 186, 262–264]. Glutamine-dependent tumors are generally less detectable than glucose-dependent under FDG-PET imaging, but could be detected under glutamine-based PET imaging [265]. Glutamine targeting should have therapeutic benefit against those tumors that depend on glutamine for growth and survival. We found that the highly metastatic VM-M3 tumor cells are dependent primarily on availability of glutamine for growth and ability to spread systemically [108]. The glutaminase inhibitor DON (6-diazo-5-oxo-L-norleucine) has shown therapeutic benefit in the clinic, as long as toxicity can be managed [186, 266]. DON could work best when combined with inhibitors of glycolysis such as lonidamine [186]. In addition to DON, other glutamine inhibitors ((bis-2-(5-phenylacetamido-1,2, 4-thiadiazol-2-yl)ethyl sulfide, BPTES, or CB-839) could also be therapeutic in targeting glutamine-dependent tumors [267]. A greater attention to possible adverse effects will be needed for glutamine targeting than for glucose targeting, as glutamine is involved with several essential physiological functions especially for cells of the immune system [268, 269]. It might therefore be necessary to also periodically schedule glutamine supplementation with glutamine targeting to obtain maximum therapeutic benefit while protecting immune system function.

The VM-M3 tumor is an excellent model system for evaluating the role of glutamine as a metabolic driver of invasive and metastatic cancer. The VM-M3 tumor arose spontaneously in the brain of its syngeneic immunocompetent VM/Dk inbred mouse host [270]. The tumor was classified as a glioblastoma (GBM) based on histological appearance, invasive growth behavior in brain, and systemic metastasis when give access to extraneural sites [271–277]. The neoplastic VM-M3 tumor cells share several characteristics with mesenchymal microglia/macrophages, which are abundant in GBM and use glutamine as a major fuel [278, 279]. Although calorie restriction could partially reduce distal invasion of VM-M3 tumor cell in brain and reduce primary tumor growth in flank, CR did not prevent systemic metastasis despite causing reduction in blood glucose and elevation of ketone bodies [108, 280]. However, DON had a major effect in reducing both primary tumor size and systemic metastasis indicative of the importance of glutamine in driving this tumor [108]. A synergistic interaction was also seen when DON was combined with calorie restriction [281]. Modifications of DON scheduling, timing, and dosing would be needed to improve efficacy and reduce toxicity. In this example, CR is the press and DON is the pulse. As glutamine is a major fuel of immune cells, glutamine targeting should be effective in reducing most metastatic cancers that have characteristics of macrophages and other immune cells [121].

### Optimization of scheduling, timing, and dosing

**Fig. 2:**
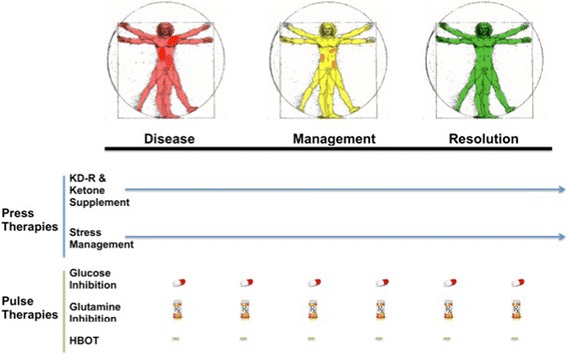
Illustration of the Press-Pulse Therapeutic Strategy for Cancer Management. The “Press-Pulse” therapeutic strategy considers cancer as a singular systemic disease regardless of the specific tissue or organ system containing invasive or metastatic tumor cells. This strategy is designed to target the glucose and glutamine dependency of tumor cells, while enhancing the metabolic efficiency in normal cells. Press therapies are designed to reduce systemic glucose availability while elevating blood levels of ketone bodies, which tumor cells cannot effectively use for energy generation. This approach pits the metabolic demands of normal cells against those of the mutated tumor cells, which are less capable than normal cells in adapting to metabolic stress from nutrient deprivation. Ketone body supplements could further reduce glucose levels while enhancing the respiratory energy metabolism in normal cells. Stress management techniques together with exercise could further stress tumor cell metabolism while improving general health. The press therapies would be designed to work synergistically with acute pulse therapies to further target glucose and glutamine metabolism. HBOT will work together with the press therapies to selectively increase oxidative stress in tumor cells. The spacing between the various pulse therapies is designed to stress tumor cell metabolism while minimizing toxicity to normal body cells. This therapeutic strategy will target the fermentation metabolism common to most tumor cells, thus gradually degrading tumor burden. The progressive color change in the Vitruvian man drawing from red (diseased with darker red spots indicative of metastatic lesions), to yellow (reduced metastasis), to green (resolution) symbolizes a gradual metabolic management and resolution of cancer. The pill symbol is indicative of glycolysis targeting that could be delivered orally. The Rx symbol is indicative of glutamine targeting that could be delivered intravenously. Pulse therapies would terminate with evidence of management or resolution while press therapies could continue under modification or adjustment (*arrow*). Optimization of dosing, timing, and scheduling of the press-pulse therapeutic strategy will facilitate the eradication of tumor cells with minimal patient toxicity. This therapeutic strategy can be used as a framework for the design of clinical trials for the majority of cancers. HBOT, hyperbaric oxygen therapy; KD-R, calorie restricted ketogenic diet

The success of the press-pulse therapeutic strategy for the metabolic management of cancer will depend on optimization of the scheduling, dosing, and timing of the various diets, drugs, and procedures used in order to achieve maximum synergistic interactions (Fig. 2). Scheduling will involve the order in which the chosen pulses are delivered to the subject while under dietary therapy. Timing will determine when and for how long the presses and pulses are given (number/day,/week,/month etc.). Dosing will identify the optimal drug dosages needed to kill tumor cells while preventing or minimizing systemic toxicity. Scheduling for each of these variables can be adjusted for the age, sex, and general health status of the subject. The strategy should degrade tumor cell populations gradually to prevent tumor lysis syndrome, which could cause excessive toxicity. Tumor imaging procedures involving FDG-PET, magnetic resonance imaging (MRI), and computed tomography perfusion (CTP), as well as analysis of serum cancer biomarkers should be helpful in assessing therapeutic success. The goal of the press-pulse therapeutic strategy is to improve progression-free and overall survival from cancer without producing adverse effects from the treatment.

## Discussion & Conclusions

Many of the current treatments used for cancer management are based on the view that cancer is a genetic disease. It is clear from the cancer death statistics that most current therapies are wanting in their ability to reduce the yearly death rate or to manage the disease without toxicity. Emerging evidence indicates that cancer is a mitochondrial metabolic disease that depends on availability of fermentable fuels for tumor cell growth and survival. Glucose and glutamine are the most abundant fermentable fuels present in the circulation and in the tumor microenvironment. The press-pulse therapeutic strategy is designed to target availability of glucose and glutamine thus starving tumor cells of their most important fuels and increasing their vulnerability to oxidative stress and apoptotic death. Low-carbohydrate, high fat-ketogenic diets coupled with glycolysis inhibitors will reduce metabolic flux through the glycolytic and pentose phosphate pathways needed for synthesis of ATP, lipids, glutathione, and nucleotides. DON and other similar glutamine inhibitors will deprive proliferating tumor cells of the glutamine needed for TCA cycle anaplerosis, and synthesis of glutathione, nucleotides, and proteins. Lysosomal targeting with chloroquine or similar drugs will reduce glucose and glutamine production following digestion of phagocytosed glycoconjugates and proteins. Glutamine targeting will require careful adjustments, however, as glutamine is a key metabolite needed for the immune system and for other physiological functions. Hyperbaric oxygen therapy combined with the calorie restricted ketogenic diet will kill tumor cells through apoptotic and anti-angiogenic mechanisms while also reducing inflammation in the tumor microenvironment and systemically. It is our view that the “Press-Pulse” paradigm is a compelling and parsimonious therapeutic strategy for effectively managing the vast majority of malignant cancers with minimal toxicity, as this approach will target the major energy pathways responsible for tumor cell growth and survival while enhancing the energetic efficiency of normal body cells and tissues.

## Abbreviations

2-DG: 2-deoxyglucose
CR: Calorie restriction
DON: 6-diazo-5-oxo-L-norleucine
FAD: Flavin adenine dinucleotide
GBM: Glioblastoma multiforme
GKI: Glucose Ketone Index
HBOT: Hyperbaric oxygen therapy
KD-R: Restricted Ketogenic Diet
NAD: Nicotinamide adenine dinucleotide
ROS: Reactive Oxygen Species
SLP: Substrate level phosphorylation
TCA: Tricarboxylic acid
